# A multicenter, randomized study of decitabine as epigenetic priming with induction chemotherapy in children with AML

**DOI:** 10.1186/s13148-017-0411-x

**Published:** 2017-10-05

**Authors:** Lia Gore, Timothy J. Triche, Jason E. Farrar, Daniel Wai, Christophe Legendre, Gerald C. Gooden, Winnie S. Liang, John Carpten, David Lee, Frank Alvaro, Margaret E. Macy, Carola Arndt, Philip Barnette, Todd Cooper, Laura Martin, Aru Narendran, Jessica Pollard, Soheil Meshinchi, Jessica Boklan, Robert J. Arceci, Bodour Salhia

**Affiliations:** 1 Children’s Hospital Colorado and University of Colorado School of Medicine, Aurora, CO USA; 2 Department of Translational Genomics and Norris Comprehensive Cancer Center, Jane Anne Nohl Division of Hematology, Keck School Medicine of University of Southern California, Los Angeles, CA USA; 3 Arkansas Children’s Research Institute and University of Arkansas for Medical Sciences, Little Rock, AR USA; 4 Ron Matricaria Institute of Molecular Medicine, Phoenix, AZ USA; 5 Translational Genomics Research Institute, Phoenix, AZ USA; 6 Sidney Kimmel Comprehensive Cancer Center at Johns Hopkins’ University, Baltimore, MD USA; 7 John Hunter Hospital, New Lambton Heights, New South Wales Australia; 8 Mayo Clinic, Rochester, MN USA; 9 Primary Children’s Medical Center and the University of Utah, Salt Lake City, UT USA; 10 Children’s Healthcare of Atlanta, Atlanta, GA USA; 11 Nationwide Children’s Hospital, Columbus, OH USA; 12 Alberta Children’s Hospital and University of Calgary, Calgary, AB Canada; 13 Seattle Children’s Hospital, Seattle, WA USA; 14 Fred Hutchinson Cancer Research Center, Seattle, WA USA; 15 Phoenix Children’s Hospital, Phoenix, AZ USA; 16 Center for Cancer and Blood Disorders, Children’s Hospital Colorado, 13123 East 16th Av, Box B115, Aurora, CO 80045 USA

**Keywords:** AML, Epigenetics, Pediatrics, Pharmacokinetics, Pharmacodynamics, DNA methylation

## Abstract

**Background:**

Decitabine is a deoxycytidine nucleoside derivative inhibitor of DNA-methyltransferases, which has been studied extensively and is approved for myelodysplastic syndrome in adults but with less focus in children. Accordingly, we conducted a phase 1 multicenter, randomized, open-label study to evaluate decitabine pre-treatment before standard induction therapy in children with newly diagnosed AML to assess safety and tolerability and explore a number of biologic endpoints.

**Results:**

Twenty-four patients were fully assessable for all study objectives per protocol (10 in Arm A = epigenetic priming induction, 14 in Arm B = standard induction). All patients experienced neutropenia and thrombocytopenia. The most common grade 3 and 4 non-hematologic adverse events observed were gastrointestinal toxicities and hypophosphatemia. Plasma decitabine PK were similar to previously reported adult data. Overall CR/CRi was similar for the two arms. MRD negativity at end-induction was 85% in Arm A versus 67% in Arm B patients. DNA methylation measured in peripheral blood over the course of treatment tracked with blast clearance and matched marrow aspirates at day 0 and day 21. Unlike end-induction marrow analyses, promoter methylation in blood identified an apparent reversal of response in the lone treatment failure, 1 week prior to the patient’s marrow aspirate confirming non-response. Decitabine-induced effects on end-induction (day 35–43 following initiation of treatment) marrows in Arm A were reflected by changes in DNA methylation in matched paired marrow diagnostic aspirates.

**Conclusions:**

This first-in-pediatrics trial demonstrates that decitabine prior to standard combination chemotherapy is feasible and well tolerated in children with newly diagnosed AML. Pre-treatment with decitabine may represent a newer therapeutic option for pediatric AML, especially as it appears to induce important epigenetic alterations. The novel biological correlates studied in this trial offer a clinically relevant window into disease progression and remission. Additional studies are needed to definitively assess whether decitabine can enhance durability responses in children with AML.

**Trial registration:**

NCT01177540

**Electronic supplementary material:**

The online version of this article (10.1186/s13148-017-0411-x) contains supplementary material, which is available to authorized users.

## Background

Attaining complete response/remission (CR) is currently considered the essential first step in the effective treatment of acute myelogenous leukemia (AML). Historically, the most widely used induction therapy included 7 days of cytarabine plus 3 days of anthracycline (known as “7 + 3”). With this approach, 75–80% of children with AML achieve CR [1–3]. Subsequently, the addition of a third agent such as etoposide to 7 + 3 (ADE), along with expanded supportive care measures, has led to higher remission induction rates of approximately 85%. Of patients who do not attain remission, approximately one-half have resistant leukemia and a substantial proportion will die from complications of the disease or treatment. Thus, there is a need to develop new treatment strategies to improve outcomes for these patients.

Pediatric tumors have been shown to have lower mutation burdens than adult tumors, and many of these mutations occur in the plethora of known epigenetic complexes [4]. In addition, significant aberrant DNA methylation is also observed in pediatric cancers such as AML including in patients with the poorest risk sub-types [5]. These studies argue for the importance of identifying novel epigenetic therapies that target both histone and/or DNA methylation modifications. Specifically, reversal of promoter DNA hypermethylation and associated gene silencing is an attractive therapeutic approach in adult cancers. The DNA methylation inhibitors decitabine and azacitidine are efficacious for hematological neoplasms at lower, less toxic, doses [6]. Experimentally, high doses induce rapid DNA damage and cytotoxicity, which do not explain the prolonged response observed in adult patients [6]. Studies have consistently shown that transient low doses of DNA demethylating agents exert durable anti-tumor effects on hematological and epithelial tumor cells and can therefore serve as a “priming” agent [6]. Studies have demonstrated that DNA hypomethylating agents can sensitize/prime resistant cancer cells to cytotoxic agents in vitro and in vivo [7–15] and can enhance chemosensitivity of human leukemia cells to cytarabine [16]. Therefore, pre-treatment with a DNA hypomethylating agent may increase the efficacy of pediatric AML induction therapy [17]. However, to date, there are no studies demonstrating the safety, tolerability, or efficacy of decitabine in combination with conventional multi-agent chemotherapy for AML in children. We report here the first phase 1 clinical evaluation of decitabine in children with newly diagnosed AML as a feasibility study to determine the safety, tolerability, and preliminary efficacy when used as epigenetic priming agent before induction chemotherapy. In addition to assessing toxicity and morphologic remission, this study examined decitabine pharmacokinetics and minimal residual disease (MRD) impact. We also performed global DNA methylation analysis to examine how decitabine priming impacted the methylome in end-induction marrows when compared with matched diagnostic marrow baseline controls. We believe that this feasibility study was essential prior to longer-term studies assessing whether epigenetic-directed therapy in pediatric AML can lead to enhanced response rates or more durable responses.

## Methods

### Patient eligibility

Eligible patients were 1 to 16 years of age (inclusive), had histologically confirmed de novo AML with > 20% bone marrow blasts, and adequate cardiac function (defined as ejection fraction > 50% or shortening fraction > 26%). Patients with acute promyelocytic leukemia (FAB M3 subtype), symptomatic CNS involvement, white blood cell count over 100,000/μl, significant renal or hepatic disease, any prior chemotherapy or radiation therapy for AML, known HIV infection, history of CML, and congenital syndromes known to predispose to AML (for example, Down syndrome, Fanconi anemia, Kostmann syndrome, or Diamond-Blackfan anemia) were excluded.

The study protocol was approved by the institutional review boards at participating sites and was conducted in accordance with the Declaration of Helsinki, Good Clinical Practice, and all local and federal regulatory guidelines. A parent or legal guardian provided written informed consent, with patient assent as appropriate according to institutional requirements.

### Study design

This multicenter, open-label study randomized patients to one of two arms: either 5 days of decitabine followed by standard induction chemotherapy with cytarabine, daunorubicin, and etoposide (Arm A = DADE), or standard induction chemotherapy with cytarabine, daunorubicin, and etoposide without decitabine (Arm B = ADE). The trial was listed under ClinicalTrials.gov identifier NCT00943553. Twenty-five children age 1–16 years with newly diagnosed de novo AML were randomized to receive either Arm A or Arm B. Given the feasibility nature of the study, sample size was selected based on the likelihood of how many patients might be accrued in a reasonable time frame so that future studies could be planned. Patients were stratified by age group and then randomized within each stratum in a 1:1 ratio by an Interactive Voice Response System via a random number generator. Three age strata were used: 1 to < 2 years, 2–11 years, and 12–16 years, with efforts made to balance enrollment among the age groups.

All patients received one cycle of study treatment, which consisted of 15 (Arm A) or 10 (Arm B) days of chemotherapy followed by a 4-week observation period, in the absence of clinically significant disease progression, unacceptable toxicity, or patient/guardian choice to discontinue participation. Patients were not pre-medicated prior to the first dose of decitabine; however, all other supportive care measures were allowed according to institutional standards. Following the completion of the study therapy, therapy continued at the treating physician’s discretion.

Treatment was administered to patients in hospital, and hospitalization through count recovery was mandated. The dose and schedule of decitabine used in this study were known to be safe and tolerable in adults and was known to induce adequate hypomethylation [18, 19], inhibit DNA methyltransferase, and induce tumor suppressor gene activation as early as 3–5 days following initiation. Treatment included (a) decitabine 20 mg/m^2^ IV infusion for 1 h daily for 5 days (Arm A) on days 1–5; (b) age-based dosing of intrathecal cytarabine (1 to < 2 years: 30 mg; 2 to < 3 years: 50 mg; ≥ 3 years: 70 mg) at the time of diagnostic lumbar puncture or on Day 1; (c) cytarabine 100 mg/m^2^/dose (3.3 mg/kg/dose for BSA < 0.6 m^2^) slow IV push over 15 min, every 12 h for 10 days on days 1–10 (Arm B) or days 6 to 15 (Arm A); (d) daunorubicin 50 mg/m^2^ (1.67 mg/kg/dose for BSA < 0.6 m^2^) IV over 6 h for 3 days on days 1, 3, and 5 (Arm B) or days 6, 8, and 10 (Arm A); and (e) etoposide 100 mg/m^2^/dose (3.3 mg/kg/dose for BSA <0.6 m^2^) IV over 4 h for 5 days on days 1–5 (Arm B) or days 6–10 (Arm A).

Toxicity was graded according to the National Cancer Institute Common Terminology Criteria for Adverse Events (CTCAE), version 4.0 (http://ctep.cancer.gov; National Cancer Institute, Bethesda, MD). Treatment-related toxicity was defined as non-resolving grade 3 or grade 4 non-hematologic or hematologic toxicity or time to platelet recovery to ≥ 100,000/μl and neutrophil recovery to ≥ 1000/μl more than 55 days from the last day of induction chemotherapy in the absence of leukemia. Events considered by the investigator to be possibly, probably, or definitely related to decitabine were considered treatment-related toxicity. Toxicities were assessed on a continuous basis for all study participants throughout treatment and were followed until count recovery, resolution, or determination that no further improvement in toxicity would occur, as assessed by the treating investigator.

### Safety assessments

Induction mortality was defined as death occurring within 6 weeks following initial diagnosis of AML. An independent Data Safety and Monitoring Board assessed the first 12 patients enrolled. This Board remained active for continuous analyses and recommendations throughout the conduct of the study. Stopping rules were included in the protocol to ensure appropriate safety of participants and that in the event of unacceptable toxicity additional patients would not be placed at risk. All investigators had access to the primary clinical trial data.

### On-study evaluations

Required assessments included physical examinations and recording of adverse events at screening/baseline, on day 5, and at the completion of study therapy. Required hematology and serum chemistry assessments were performed on days 1, 2, 6, 7, 14, 15, and weekly thereafter. Bone marrow evaluations for morphology, MRD, and molecular analyses were performed at screening/baseline, 3–4 weeks following the completion of induction chemotherapy regardless of peripheral blood count recovery, and then as clinically indicated until count recovery. Any clinically appropriate assessment or test was allowed at the treating physician’s discretion to maintain standards of care.

### Efficacy assessments

The primary efficacy variable was CR, defined by the International Working Group 2003 criteria [20], requiring patients to have a morphologic leukemia-free state and an absolute neutrophil count of > 1000/μL and platelets of > 100,000/μL. Neither hemoglobin nor hematocrit was considered to have bearing on response although patients were required to be red blood cell transfusion independent to enroll. Secondary efficacy variables included leukemia-free survival (LFS), overall survival (OS), methylation of DNA following decitabine therapy, times to platelet and neutrophil recovery, and level of minimal residual disease at the end of induction therapy. LFS and OS were assessed on patients every 3 months until disease progression, death, or loss to follow-up. MRD analysis was performed at the post-induction therapy assessment by Difference-From-Normal (DFN) panels by flow cytometry [21]. Children with MRD by flow between 0.01 and 0.05% of normal bone marrow nucleated cells were considered negative; children above 0.05% were considered positive. The sensitivity of this method is reported to be 10^−4^ cells [21].

Unlike Leukemia-Associated ImmunoPhenotype (LAIP) panels for MRD, which succeeds in 80–85% of AML patients, DFN produces results in 100% of patients [21].

Due to the small sample size, statistical analyses were primarily descriptive.

### Pharmacokinetic evaluations

Serial blood samples (2 mL each) were drawn from all patients randomized to Arm A at pre-decitabine, 30, 60 (just prior to the end of infusion), 65, 90, 120, and 180 min after the start of decitabine infusion. A separate line was used to draw PK samples not in proximity (i.e., not the contralateral lumen of a double lumen line) to the decitabine infusion. Samples were collected in EDTA tubes containing tetrahydrouridine, a cytidine deaminase inhibitor, to prevent decitabine degradation, and were centrifuged at 4 °C within 30 min of collection. Plasma was harvested and stored frozen at − 70 to − 80 °C and shipped on dry ice for central analysis.

Pharmacokinetic parameters were calculated from plasma decitabine concentration-time data by non-compartmental methods using Phoenix WinNonlin version 6.2 (Pharsight Corporation, Mountain View, CA). The maximum plasma concentration (*C*_max_) and the time at which *C*_max_ occurred (*T*_max_) were determined by inspection of the individual data. AUC from time 0 until the last quantifiable concentration (AUC_0–tau_) was determined by the linear up-log down trapezoidal rule. The terminal phase elimination rate constant (*K*_el_) was estimated from the slope of the concentration-time data during the log-linear terminal phase using least square regression analysis. The terminal phase elimination half-life (*t*_1/2_) was calculated using the formula 0.693/*K*_el_. The AUC-time curve from 0 to infinity (AUC_0-infinity_) was computed as AUC_0-t_ plus the extrapolation from the last quantifiable concentration, *C*_t_, to infinity using the formula C_t_/*K*_el_. Total body clearance (CLp) was calculated by the formula Dose/AUC_0-infinity_. The volume of distribution at steady state (*V*_dss_) was calculated using the formula CL_p_ × MRT. Area under the first moment curve (AUMC) was determined using the linear trapezoidal rule to calculate AUMC_0-tau_ and extrapolated to infinity as AUMC_0-tau_ + *t* × *C*_t_/(*K*_el_) [2]. The formula used to determine mean residence time (MRT) was [AUMC/AUC_0-infinity_ − tau/2], where tau is the duration of infusion.

### DNA methylation analysis

Bone marrow and blood samples were obtained from all patients at baseline and at completion of induction therapy. In addition, blood samples were also collected on days 7 and 14. DNA was extracted from marrow or peripheral blood lymphocytes (buffy coat) using Qiagen’s AllPrep kit from samples enriched for leukemic blasts by standard Ficoll separation. Global DNA methylation was evaluated using the Infinium® Human Methylation450® BeadChip Array according to the manufacturer’s protocol (Illumina, San Diego, CA) and as previously described [22–24]. A total of 18 paired patient samples with both diagnostic and remission bone marrows (9 pairs from Arm A and 9 pairs from Arm B, totaling 36 samples) were used for DNA methylation analyses. In addition, peripheral blood DNA from all time points was also analyzed. DNA methylation levels for each CpG residue are presented as *β* values, estimating the ratio of the methylated signal intensity over the sum of the methylated and unmethylated intensities at each locus. The average *β* value reports a methylation signal ranging from 0 to 1, representing completely unmethylated to completely methylated values, respectively. DNA methylation data were preprocessed using the Illumina Methylation Analyzer (IMA; doi: 10·1093/bioinformatics/bts013), including background and probe design corrections, quantile normalization, and logit transformation. Loci with detection *p* values > 0.05 in 25% of samples, on sex chromosomes, or within 10 bp of putative SNPs were removed from analysis. Differential methylation analysis was performed by IMA. A paired Wilcoxon rank test was conducted to compare end-induction marrows with diagnostic marrows within each arm. Probes with *p* < 0.05 having group-wise differences in *β* values of at least 0.15 [23, 24] were considered statistically significant and differentially methylated. Differentially methylated loci were visualized on a heat map, and separation of groups was assessed by hierarchical cluster analysis using Manhattan distance and Ward’s method. Unsupervised clustering was also performed on the top 0.1% most variable probes by standard deviation. The DNA methylation data discussed here were deposited in NCBI Gene Expression Omnibus Database and are accessible through GEO Series accession number GSE78963.

### Pathway analysis

Gene lists derived from DNA methylation analysis were uploaded into IPA (Ingenuity® Systems, Redwood City, CA), and the Core Analysis workflow was run with default parameters. Core Analysis provides an assessment of significantly altered pathways, molecular networks, and biological processes.

## Results

### Patients

Twenty-five patients, aged 1–16 years (median 8.0 years) with WBC at diagnosis ranging from 1.19 to 58.09 × 10^3^/μL were randomized between March and November 2011. Two patients did not receive the full induction regimen due to toxicity, one in Arm A who discontinued study participation on day 6 (described more fully below), and one in Arm B, whose family declined completion of chemotherapy due to low level (grade 1 and 2) toxicities but allowed all study assessments to be completed. As shown in Table 1, 24 were fully assessable for all study objectives per protocol (10 of 11 in Arm A, and all 14 in Arm B). Three patients had confirmed FLT3 internal tandem duplications, all with an allelic ratio of ≥ 0.5 and one had a FLT3 D835 point mutation. Two patients had NPM1 mutations and two patients had CEBPA mutations. No patients had mutations of TET2, IDH1, IDH2, or C-CBL exons 8 or 9. One patient each had a KIT exon 8 (N822K) and 17 (D816H) mutation. Three patients had WT1 exon 7 mutations and one patient had a WT1 exon 9 mutation. On relative dose intensity analysis, patients received 99–100% of the intended doses of decitabine, daunorubicin, and etoposide and 84% of the intended doses of cytarabine.

**Table 1:** Patient characteristics (by arm and overall)

Characteristic	All Patients	…in Arm A	…in Arm B
Consented	25 (100%)	11 (44%)	14 (56%)
Randomized and treated	25 (100%)	11 (44%)	14 (56%)
Evaluable for all study endpoints	24 (96%)	10 (40%)	14 (56%)
Sex:			
Male	12 (48%)	7 (58%)	5 (42%)
Female	13 (52%)	4 (31%)	9 (69%)
Age, years			
Median (range)	8.0 (1–16)	8 (2–16)	7.5 (1–16)
Race:			
White	20 (80%)	8 (32%)	12 (48%)
African-American	1 (4%)	1 (4%)	0 (0%)
Asian	1 (4%)	0 (0%)	1 (4%)
American Indian or Alaska native	1 (4%)	1 (4%)	0 (0%)
Other	2 (8%)	1 (4%)	1 (4%)
Ethnicity, Hispanic	6 (24.0)		
Ethnicity, not Hispanic or Latino	19 (76.0)		
Cytogenetic risk groups:			
Favorable	4 (8%)	1 (4%)	3 (12%)
Intermediate	17 (68%)	6 (24%)	11 (44%)
Unfavorable	4 (16%)	4 (16%)	0 (0%)
Cytogenetics:			
Normal	7 (28%)	3 (12%)	4 (16%)
t(8;21)	4 (16%)	1 (4%)	3 (12%)
11q23	9 (36%)	5 (20%)	4 (16%)
Other	5 (20%)	2 (8%)	3 (12%)
Location:			
Alberta (Canada)	1 (4%)	1 (4%)	0 (0%)
Children’s Hospital Central California	1 (4%)	0 (0%)	1 (4%)
Denver	8 (32%)	5 (20%)	3 (12%)
Emory	1 (4%)	0 (0%)	1 (4%)
Johns Hopkins	5 (20%)	5 (20%)	0 (0%)
John Hunter Hospital (Australia)	2 (8%)	0 (0%)	2 (8%)
Mayo Clinic	1 (4%)	1 (4%)	0 (0%)
Nationwide Children’s Hospital	1 (4%)	0 (0%)	1 (4%)
Phoenix Children’s Hospital	1 (4%)	0 (0%)	1 (4%)
Primary Children’s Hospital	1 (4%)	0 (0%)	1 (4%)
Royal Children’s Hospital (Australia)	3 (12%)	1 (4%)	2 (8%)

### Toxicity

Treatment-emergent AEs are summarized in Table 2. The most common grade 3 and grade 4 AEs were hematologic, including WBC decreased, anemia, platelet count, and neutrophil count decreased. Colitis (*n* = 2), anorexia (*n* = 3), hypophosphatemia (*n* = 2), and hypokalemia (*n* = 3) were also noted. One patient in Arm A experienced colonic perforation on day 6 due to leukemic infiltration of the bowel wall that led to study discontinuation. Two patients in Arm A died 6 months after completion of induction therapy; one of necrotic bowel and *Pseudomonas* sepsis, and one of multisystem organ failure. The latter patient died 5 months after study treatment as a complication of stem cell transplantation. Neither death was attributed to decitabine nor to the chemotherapy regimen received during study participation.

**Table 2:** Grade 3 and grade 4 treatment-emergent adverse events (TEAEs) reported in treated patients fully assessable for all study endpoints, as assessed by the Common Terminology Criteria for Adverse Events, version 4.0

	Decitabine+ Chemotherapy		Chemotherapy only	
Adverse event	Grade 3 *n* (%)	Grade 4 *n* (%)	Grade 3 *n* (%)	Grade 4 *n* (%)
Any Grade 3 or Grade 4 TEAEs	7 (87.5)	7 (87.5)	7 (77.8)	7 (77.8)
Blood and lymphatic system disorders				
Anemia	6 (75.0)	2 (25.0)	4 (44.4)	1 (11.1)
Febrile neutropenia	1 (12.5)	0	4 (44.4)	0
Other: Neutropenia	1 (12.3)	3 (37.5)	1 (11.1)	1 (11.1)
Other: Thrombocytopenia	4 (50.0)	4 (50.0)	0	2 (22.2)
Gastrointestinal disorders				
Colitis	2 (25.0)	0	2 (25.0)	0
Investigations				
Neutrophil count decreased	0	0	1 (11.1)	3 (33.3)
Platelet count decreased	0	1 (12.5)	2 (22.2)	3 (33.3)
White blood cell count decreased	6 (75.0)	6 (75.0)	3 (33.3)	5 (55.6)
Metabolism and nutrition disorders				
Decreased appetite	3 (37.5)	0	0	0
Hypokalemia	3 (37.5)	0	1 (11.1)	0
Hypophosphatemia	2 (25.0)	0	0	0

Time to blood count recovery in the two study arms was assessed by Kaplan-Meier Analysis and suggested a slightly but non-statistically significant trend toward longer time to recovery of ANC and platelets for patients treated with decitabine compared to those who received standard ADE chemotherapy alone. Median time to platelet count ≥ 100,000/mm3 for Arm A (decitabine) was 21 (range 2–31) days and for Arm B was 12 (range 2–24) days. Median time to absolute neutrophil count ≥ 1000/mm3 was 21 days for Arm A (range 2–43 days) and for Arm B, 17.5 days (range 9–39 days). There were also no statistically significant differences between time to ANC or platelet recovery for the 1st quartile (95% CI) and 3rd quartile (95% CI).

### Pharmacokinetics

**Fig. 1:**
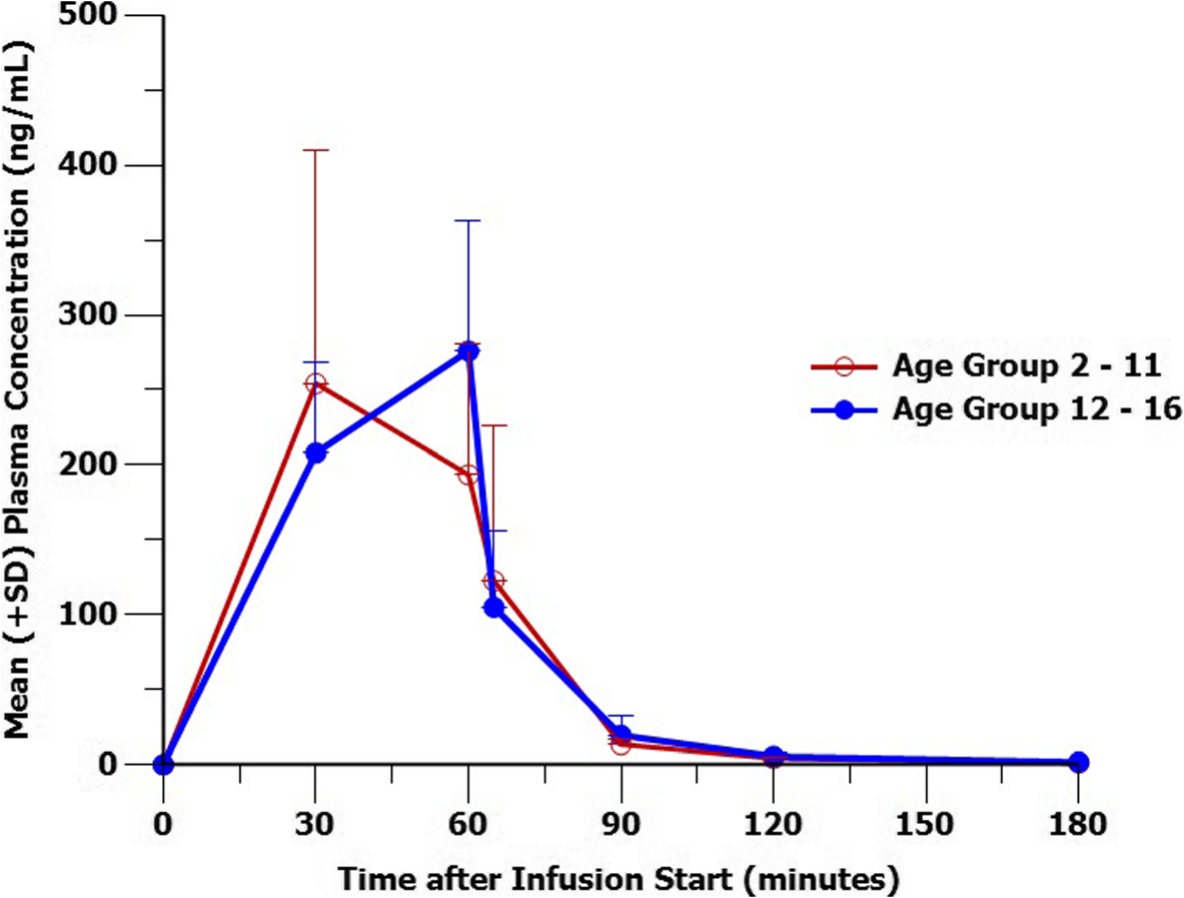
Mean decitabine blood concentration-time profile measured in whole blood by age group

Plasma concentrations of decitabine were quantifiable in all patients up to the last time point of 180 min. Post-infusion, plasma concentrations declined in a bi-exponential manner (Fig. 1). Selected PK parameters of decitabine in subjects overall are shown in Table 3. The overall mean (standard deviation) PK parameters for the decitabine-treated patients were *C*_max_, 294 (104) ng/mL; AUC_0-∞_, 214(72.4) ng h/mL; CL, 128(92.3) L/h; Vd_ss_, 45.5(41.1) L; *t*_½_, 0.453(0.0804) h; and *t*_max_, 0.831 h (0.253). Estimated PK values for a 70-kg adult male receiving a 1 h decitabine 20 mg/m^2^ infusion and merged data from prior publications of decitabine in adults [25–27] at 15 and 20 mg/m^2^ dosing are shown for reference. The mean exposure to decitabine, as measured by *C*_max_ and AUC, was similar in patients aged 12–16 years compared with those aged 2–11 years as shown, and similar to those previously reported in children in adults [25–27] with the acknowledgement that plasma half-life of decitabine in children may be shorter due to their higher activity of cytidine deaminase in the liver and spleen [28, 29]. However, inter-patient variability in this study was high.

**Table 3:** Mean pharmacokinetic parameters of decitabine on day 5 of treatment—overall and by age group, pharmacokinetic analysis population

	Age group (years)		Arm A	Comparator	Combined data from prior studies of decitabine in adults and children
Pharmacokinetic parameter	2–11 (*N* = 7)	12–16 (*N* = 4)	Total (*N* = 11)	70-kg adult male^a^	
*C*_max_ (ng/mL)	286 (131)	307 (36·9)	294 (104)	107	64·8–77·0
*t*_max_ (h)	0·803 (0·272)	0·88 (0·243)	0·831 (0·253)	NR	End of infusion
*t*_½_ (h)	0·458 (0·0777)	0·446 (0·0967)	0·453 (0·0804)	1·14	0·33–0·78
AUC_0-t_ (ng h/mL)	211 (90·0)	218 (35·1)	214 (72·4)	NR	NR
AUC_0-∞_ (ng h/mL)	212 (90·0)	219 (35·7)	215 (72·5)	580^b^	152–163
CL (L/h)	110 (113)	161 (23·9)	128 (92·3)	298	125–132
Vd_ss_ (L)	40·7 (52·0)	54·1 (9·67)	45·5 (41·1)	116	36·88–52·47

All values presented as mean ± SD. Standard deviation cannot be calculated where *n* = 2

*AUC* area under the concentration-time curve, *CL* total body clearance, *C*_*max*_ maximum concentration, *NR* not reported, *t*_*½*_ half-life, *t*_*max*_ time to *C*_max_, *Vd*_*ss*_ volume of distribution at steady-state concentrations

^a^Data based on population pharmacokinetic analysis provided by Eisai Inc.

^b^Cumulative AUC value over entire 5-day dosing period. Single-day AUC value = 116 ng h/mL

### Anti-leukemic response

Morphologic CRs and CRs with incomplete count recovery (CRi) rates were similar in both treatment arms: 100% CR/CRi in Arm A (decitabine) and 92% CR/CRi in Arm B (control). The patient who discontinued study participation on day 6 after receiving all decitabine doses and only one dose of cytarabine remained in a complete remission for 2 months without any further leukemia-directed treatment. She eventually resumed standard therapy 2 months later and remains in CR 56+ months later. Disease-free survival (DFS) at 24 months was 43% in Arm B and 50% of evaluable patients in Arm A. This is the last time point available for all patient outcomes reporting.

MRD analysis by multiparameter flow cytometry at end induction showed no difference between patients receiving Arm A or Arm B chemotherapy. Nine patients in each arm had assessable MRD at the defined end induction time point. Seven of nine patients in Arm A and six of nine patients in Arm B were MRD negative at the protocol defined cutoff of 0.05% or less. Two of nine patients in Arm A and three of nine patients in Arm B were MRD positive, ranging from 0.06% to greater than 10% detectable disease (Additional file 1: Table S1).

### DNA methylation analysis reveals DAC-induced changes

Quantitative DNA methylation analyses revealed global changes in methylation following decitabine priming. Diagnostic and end-induction marrows were analyzed in nine patients in each Arm (Additional file 2: Figure S1). Paired differential methylation analysis of end-induction marrows to patient matched screening marrows revealed 6990 differentially methylated CpG loci (DML) encompassing 2518 genes in Arm A compared to only 1090 DML (539 genes) in Arm B (Additional file 3: Tables S2A-B). Only DML in Arm A (*n* = 4597) survived false discovery *p* value correction. Of all DML in Arm A, 4134 were hypomethylated and 2856 were hypermethylated. In Arm B, 785 DML were hypomethylated and 305 were hypermethylated. There were 795 DML (438 genes) common to both arms. Although about 80% of genes altered by DNA methylation in Arm B were common to Arm A, there were significantly more probes altered for a given gene in Arm A. Moreover, 78% of hypomethylated probes in Arm B were common with Arm A, compared with 56% of hypermethylated probes common between treatment arms. The median delta-beta values for Arms A and B were − 0.27 and − 0.28, respectively, indicating modest overall hypomethylation induced by either treatment regimen at the specified delta-beta cutoff. Forty-one percent of DML were hypermethylated after decitabine therapy compared with 28% after chemotherapy only. Regional and functional CpG distribution of DML after therapy in both treatment arms was also examined. Functional distribution relates CpG position to transcription start sites (TSS) − 200 to − 1500 bp, 5′ untranslated region (UTR), and exon 1 for coding genes as well as gene bodies. In both treatment arms, gene body hypermethylation was the most frequent change, followed by gene body hypomethylation and TSS 200 hypomethylation (Additional file 4: Figure S2). Regional distribution of DML was assessed based on proximity to the closest CpG island. In addition to CpG islands, shores are 0–2 kb from CpG islands, shelves are 2–4 kb away, and open sea regions are isolated loci without a designation. CpG island hypomethylation occurred in greater than 68% of DML in both groups. Hypermethylation occurred most prominently in open sea regions and to a greater degree in Arm A patients compared with those in Arm B receiving chemotherapy alone (Additional file 5: Figure S3).

**Fig. 2:**
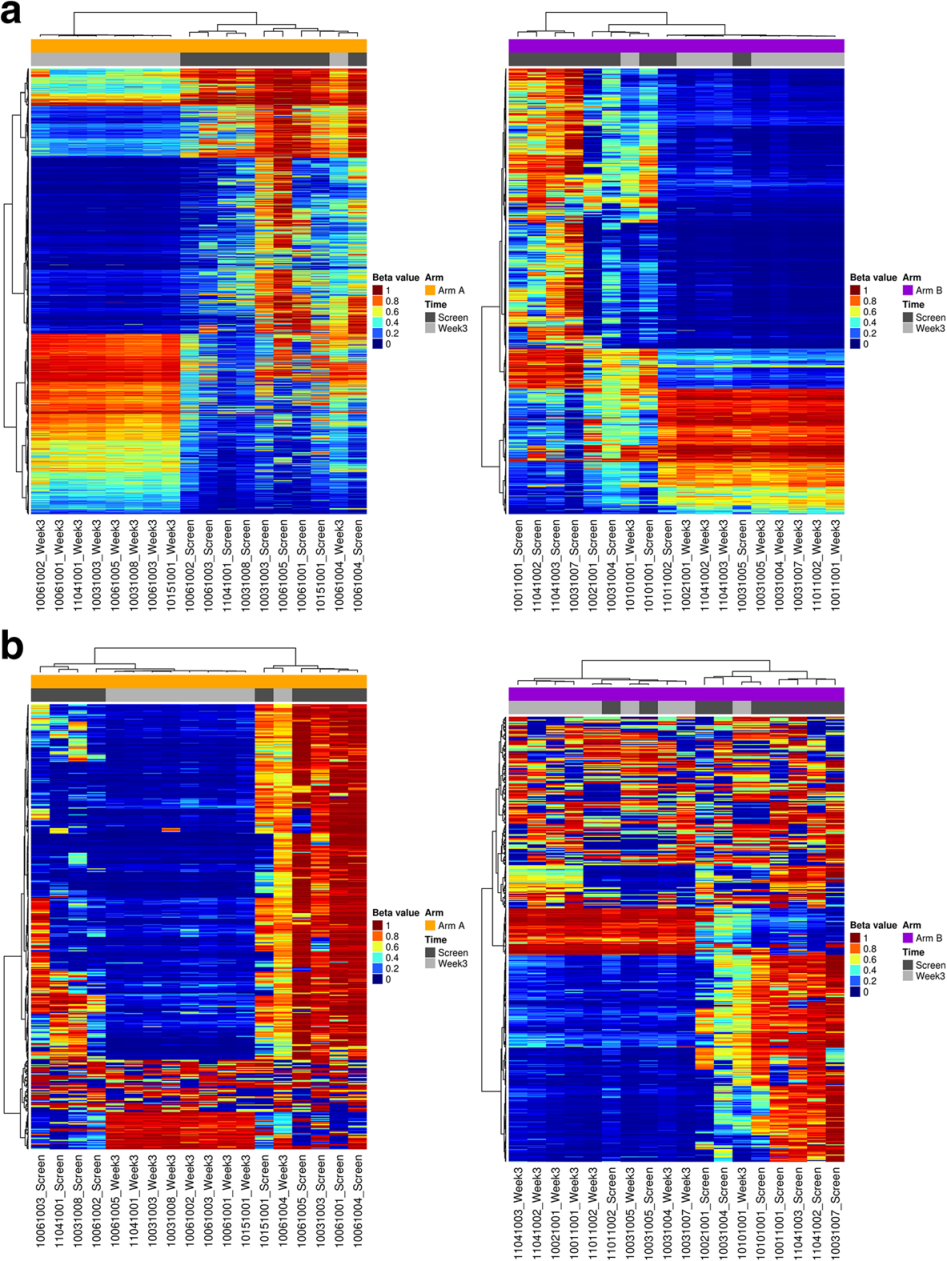
Hierarchical clustering of differentially methylated loci (DML) in Arm A (decitabine + chemotherapy) and Arm B (chemotherapy alone). **a** unsupervised clustering analysis of 6990 DML in Arm A (left panel) and 1090 DML in Arm B (right panel) revealed separation of end of induction recovering marrows at week 3 from screening marrows. **b** Unsupervised hierarchical clustering of the top 0.1% most variable loci by standard deviation also separated screening marrows from end of induction recovery marrows at week 3

Unsupervised clustering analysis of DML for both treatment arms demonstrated strong separation of screening and end-induction marrows except for one sample pair in Arm A and two sample pairs in Arm B (Fig. 2a). In these cases, pre- and post-treatment samples co-clustered with its matching sample. One case in Arm A and Arm B clustered with diagnostic marrows, suggesting the marrow was possibly unaffected by therapy, and indeed, the sample in Arm A (1006_1004) was from a patient with stable disease. Overall, these data indicate that decitabine therapy has an epigenetic effect on the recovering end-induction marrow in AML. This was evident when compared with Arm B samples, where DNA methylation was more heterogeneous after standard chemotherapy treatment.

**Fig. 3:**
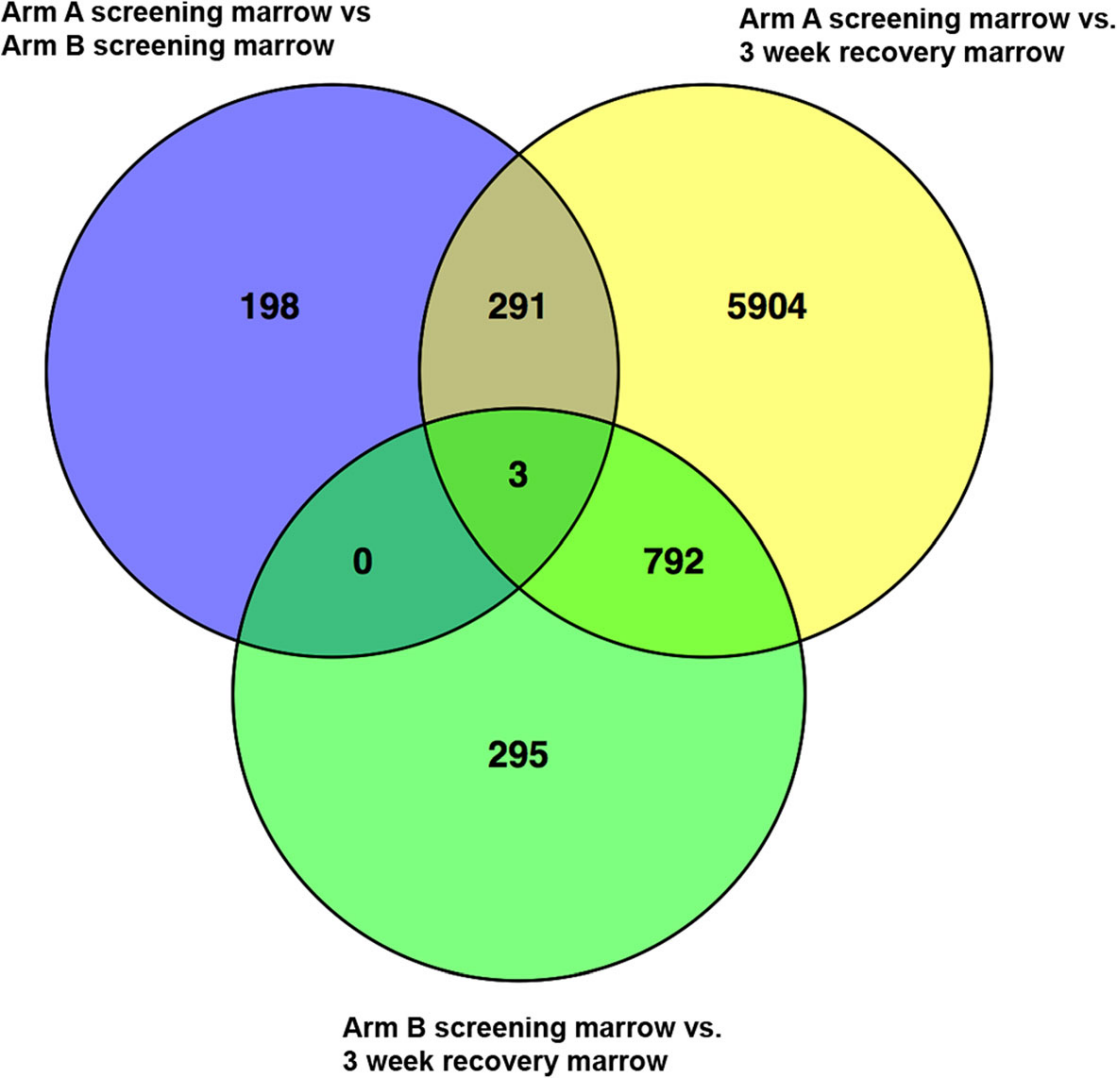
Overlap of differentially methylated loci between arms and time points in Arm A (DADE), Arm B (ADE), and screening vs. recovery marrow aspirates. Screening marrows for samples in Arm A and Arm B are also compared and demonstrate little intrinsic bias between groups

To further assess the changes in recovering marrows in both arms, we performed an unsupervised clustering analysis of the top 0.1% most variable CpG probes (~ 430 probes) by standard deviation (Fig. 2b). These data confirmed that the end-induction recovering marrows were distinctive from screening marrows and more consistent across samples in the decitabine-treated arm compared with those in the control arm (Fig. 2b). Clustering all DML for Arm A and Arm B together and the top 0.1% most variable probes (Additional file 6: Figure S4) demonstrated separation of week 3 marrows from screening marrows**.** A clear separation of the two arms was not evident due to the fact that about 73% of loci in Arm B were common to Arm A (Fig. 3). To ensure that samples were not molecularly different at screening between the arms, we performed the above analyses comparing screening marrows in Arm A with Arm B and observed only 492 DML. Of these, 291 were common to the Arm A DML list, while 3 DML were common to both Arm A and B comparisons and 0 DML were common to Arm B DML (Fig. 3).

**Fig. 4:**
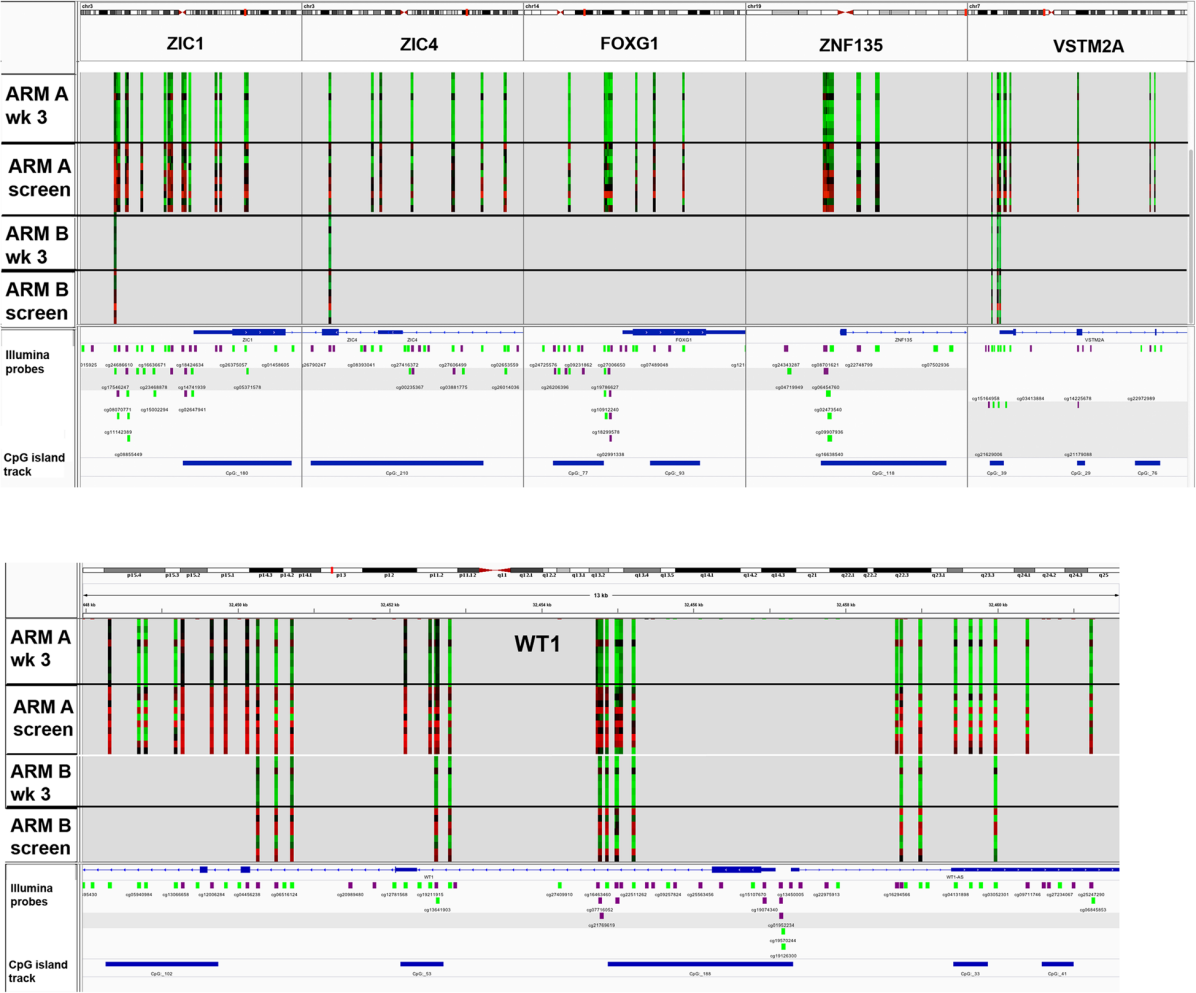
Integrated Genomic Viewer snapshot of differentially methylated genes affected by hypomethylation in response to decitabine therapy. Vertical heatmaps represent significantly differentially methylated (*p* value < 0.05) probes in the six genes illustrated. Each row on the heatmap represents a unique sample. Many more probes were differentially methylated in Arm A (decitabine+ chemotherapy) compared with Arm B (chemotherapy alone) for the probes shown. Hypomethylation (green) in response to decitabine (Arm A) is evident in end of induction recovery marrows (week 3) compared with diagnostic marrows (screen). Array avg *β* values are represented in the heatmap. Scale ranges from 0 to 1, where 0 is unmethylated (green) and 1 is fully methylated (red). Tracks shown gene, CpG 450 K probe, and CpG island

**Fig. 5:**
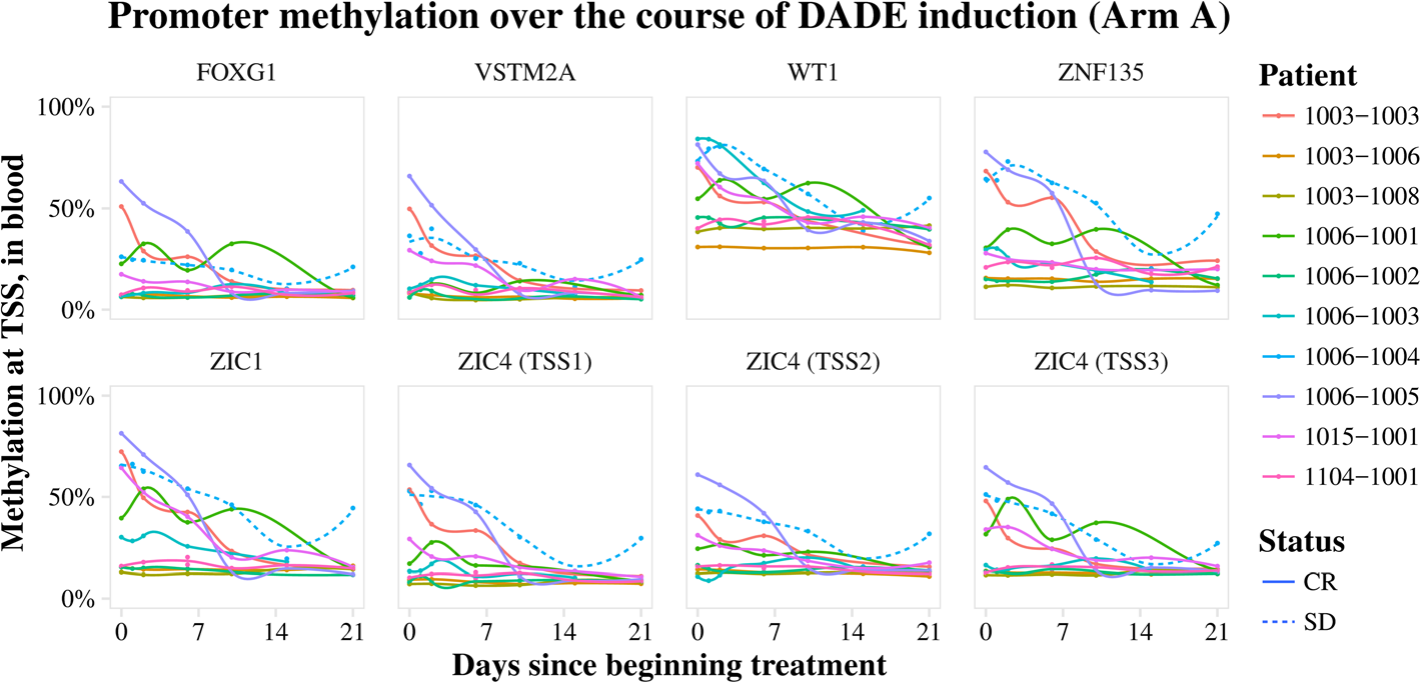
Time-course collection of peripheral blood samples in Arm A reveals consistent decreases in promoter methylation at relevant transcription start sites over treatment in all responders, as well as a reversal of this decrease in the sole non-responsive patient (1006-1004). A distinct uptick in the patient’s promoter methylation from day 14 to 21 is noted, which corresponded clinically to the patient’s disease progression

Among the genes, most prevalently hypomethylated in Arm A were FOXG1, VSTM2A, WT1, ZNF135, ZIC1, and ZIC4 (Fig. 4), which may potentially be used to measure decitabine activity. In addition, time-dependent promoter hypomethylation of these genes also occurred in peripheral blood lymphocytes (Fig. 5), confirming their significance as potential biomarkers of decitabine response. Most notably, recovery of promoter methylation in peripheral blood was seen in a patient with stable disease and whose recovering marrow co-clustered with diagnostic marrow, hinting at signs of preliminary efficacy. The data point to the potential utility of these genes as biomarkers of minimal residual disease in patients treated with decitabine.

Table 4 shows DNA methylation changes in several key biological pathways potentially important to response to decitabine and chemotherapy. The top canonical pathways in IPA for Arm A DML included gene alterations affecting mostly neuronal signaling such as neuropathic pain signaling and glutamate receptor signaling (Table 4). In Arm B, the top IPA canonical pathways included DML affecting embryonic stem cell signaling and Rho GTPase signaling (Table 4).

**Table 4:** Ingenuity Pathway Analysis of differentially methylated genes

	*p* value	Overlap *n* (%)
Arm A top canonical pathways		
Neuropathic pain signaling in doral horn neurons	7.5e−12	35/100 (35.0%)
Glutamate receptor signaling	2.45e−11	25/57 (43.9%)
Amyotrophic lateral sclerosis signaling	2.05e−09	31/98 (31.6%)
Synaptic long-term potentiation	1.00e−06	30/119 (25.2%
Hepatic fibrosis/hepatic stellate cell activation	1.01e−06	40/183 (21.9%)
Arm B top canonical pathways		
Transcriptional regulatory network in embryonic stem cells	2.68e−04	4/40 (10.0%)
Thrombin signaling	7.94e−04	7/191 (3.7%)
GPCR-mediated integration of enteroendocrine signaling Exemplified by an L Cell	2.36e−03	4/71 (5.6%)
Signaling by Rho Family GTPases	2.54e−03	7/234 (3.0%)
CXCR4 signaling	7.03e−03	5/152 (3.3%)

Differentially methylated genes for Arm A (decitabine + chemotherapy; *n* = 2518) and Arm B (chemotherapy alone; *n* = 539) were entered into the core pathway analysis option. Top 5 canonical pathways are shown along with a significant *p* value and the number of genes in each list belonging to the pathway

## Discussion

This first-in-pediatric randomized trial of epigenetic priming in children with newly diagnosed AML demonstrated safety and tolerability and establishes the feasibility required to develop future trials for assessing enhancement and durability of response. The results trended toward non-inferiority of morphologic and immunophenotypic response, although the small sample size limited statistical analyses. There was also evidence of decitabine-induced effects in end-induction bone marrow aspirates compared with those obtained at diagnosis. Children treated with decitabine as a single agent for 5 days prior to conventional cytotoxic therapy did not have rapid progression of their leukemic burden during the pre-phase, further supporting the feasibility and safety of this approach. Based on these results, a priming dose of 20 mg/m^2^ daily for 5 days could be considered for further testing.

Most non-hematologic AEs reported in this study were mild to moderate in severity, and the safety profile of decitabine in children with AML was consistent with that seen in adults [30]. In the adult study, Scandura and colleagues evaluated a different backbone chemotherapy regimen than the one used in this trial; however, their study population also included patients with less than favorable risk factors and showed that decitabine induced DNA hypomethylation and complete responses in a high percent of patients. No previously unreported decitabine toxicities were observed in pediatric patients. Drug-related hematologic toxicity, anorexia, and asymptomatic grade 3 hypokalemia and hypophosphatemia were slightly more common in decitabine-treated patients.

This trial was not powered to detect a difference in response between the two arms, and treatment with decitabine prior to standard induction therapy resulted in a similar morphological response compared to standard induction therapy. Of note, there were more high-risk cytogenetic patients in the decitabine arm (4 versus 0), which may indicate a benefit for decitabine priming in these patients. Patients in this study were generally representative of children with de novo childhood AML in regard to age, gender, and biologic features; however, there were slightly more patients with *WT1* and *CEBP*A mutations than previously reported. There was a non-significant trend toward a longer time to recover neutrophil and platelet counts in the decitabine-treated patients, but the 95% confidence intervals were overlapping in all analyses, and the total sample number was small. No AEs or SAEs were noted as a result of delays in count recovery. These results suggest that exposure to decitabine may have important implications for sensitizing potentially resistant leukemic clones to cytotoxic chemotherapy, resulting in deeper remissions predictive of more favorable outcomes. Larger randomized studies are needed to confirm these findings.

Notwithstanding the fact that the cellular composition of remission marrows differs from that of screening marrows, alterations in DNA methylation patterns in end-induction bone marrow between the two groups of patients suggest important consequences of exposure to decitabine priming on hematopoietic recovery after exposure to intensive chemotherapy. Our data suggest that while chemotherapy alone may have an effect on DNA methylation, the effect is clearly augmented by the addition of decitabine via epigenetic changes that may impact both leukemic, normal hematopoietic progenitors, as well as bone-marrow stromal cells. Additionally, an increased percentage of DMLs were hypermethylated in patients receiving decitabine compared to those receiving chemotherapy alone, suggesting that decitabine has an effect beyond DNA demethylation.

Furthermore, the data suggest that decitabine therapy can be used to measure patient response to therapy by assessing DNA methylation status of specific promoter regions non-invasively in blood. MRD data did not differ between patients in the different arms, but whether or not DNA methylation assessment can serve as a more predictive and sensitive approach to measuring MRD/patient response warrants further investigation. Pathway analysis of differentially methylated genes revealed a number of pathways implicated to neuronal signaling in the decitabine-treated arm only. While the implications of neuronal signaling are not currently clear, we postulate it must be related to the bone marrow niche post-treatment since no dorsal root ganglia or other neuronal tissue exist in the marrow. Presumably, this could be due to ion channel currents that play an important role in bone-marrow-derived mesenchymal stem cells and hematopoietic progenitors [31]. Our observations further suggest that monitoring changes in normal progenitors may be important in understanding the short- and longer-term consequences of exposure to methyltransferase inhibitors on malignant and normal bone marrow progenitors.

## Conclusions

The toxicity and PK results observed in the patients in this study suggest that decitabine can be safely combined with standard doses and schedules of anticancer agents in children with newly diagnosed AML. Furthermore, our data suggest that this regimen alters DNA methylation compared to ADE chemotherapy alone, and patients treated with decitabine could have minimal residual disease measured by assessing DNA methylation status of specific promoter regions. Preclinical studies have shown additive or synergistic activity when decitabine is combined with a variety of other anticancer therapies [32–35], and results from trials such as this provide further evidence of feasibility, safety, and possible strategies for larger randomized trials in patients with newly diagnosed or recurrent/refractory leukemia, as well as in the minimal disease state during post-remission follow-up. No excess or unexpected toxicities were seen. The most common drug-related grade 3 or grade 4 AEs were hematologic and PK/PD were as expected. Complete remission rates were similar. Patients treated with decitabine prior to conventional chemotherapy had distinct changes in DNA methylation, which may be of interest for further mechanistic study. In conclusion, epigenetic therapy with decitabine is safe for use in children, and the clinical findings together with molecular correlative studies suggest that there may be early signs of enhanced efficacy. However, further studies are needed to definitively determine the long-term patient outcomes of decitabine priming in children with AML.

## Additional files

Additional file 1: Table S1. Breakdown of MRD data for study participants. (DOCX 10 kb)

Additional file 2: Figure S1. Schema of sample analysis workflow. (TIFF 269 kb)

Additional file 3: Table S2A. Differentially Methylated Loci comparing week 3 marrows with screening marrows in Arm A. (XLSX 649 kb)

Additional file 4: Figure S2. Distribution of differentially methylated loci (DML) according to functional CpG contextual distribution in Arms A (decitabine + chemotherapy) and B (chemotherapy alone). Pie charts demonstrate the frequency by which hyper or hypomethylated loci are distributed according to their functional position. (TIFF 443 kb)

Additional file 5: Figure S3. Distribution of differentially methylated loci (DML) according to CpG Island contextual distribution in Arms A (decitabine + chemotherapy) and B (chemotherapy alone). Pie charts demonstrate the frequency by which hyper or hypomethylated loci are distributed according to their proximity of CpG islands. (TIFF 365 kb)

Additional file 6: Figure S4. Left Panel: Unsupervised hierarchical clustering of the union of differentially methylated loci (DML) for all samples in Arm A (decitabine + chemotherapy) and Arm B (chemotherapy alone). Right Panel: Unsupervised hierarchical clustering of the top 0.1% most variable loci by standard deviation for all samples in both arms. (PNG 619 kb)

## Abbreviations

ADE: Cytarabine/daunorubicin/etoposide chemotherapy regimen
AML: Acute myelogenous leukemia
AUC: Area under the curve
BSA: Body surface area
C max: Maximum plasma concentration
CML: Chronic myelogenous leukemia
CNS: Central nervous system
CR: Complete remission
CR: Complete remission with incomplete count recovery
DADE: Decitabine plus daunorubicin/cytarabine/etoposide chemotherapy regimen
DFN: Disease from normal
DML: Differentially methylated loci
DNA: Deoxyribonucleic acid
FAB: French-American-British classification
HIV: Human immunodeficiency virus
LAIP: Leukemia-Associated ImmunoPhenotype
LFS: Leukemia-free survival
MRD: Minimal residual disease
OS: Overall survival
PD: Pharmacodynamics
PK: Pharmacokinetics
RNA: Ribonucleic acid
Tmax: Time to maximum plasma concentration
