# Supplementary material for: A multicenter, randomized study of decitabine as epigenetic priming with induction chemotherapy in children with AML
Source: Clin Epigenetics. 2017 Oct 5;9:108. doi: 10.1186/s13148-017-0411-x (PMC5629751; doi:10.1186/s13148-017-0411-x)
Supplement: Supplementary file 1 — Breakdown of MRD data for study participants. (DOCX 10 kb) [file 13148_2017_411_MOESM1_ESM.docx]

|  | **Arm A (DADE chemotherapy)** | **Arm B (standard ADE chemotherapy)** |
| --- | --- | --- |
| Number of patients MRD negative at end induction  (range of MRD =  0 to <0.02%) | 7 | 6 |
| Number of Patients MRD positive at end induction  (range of MRD =  0.06 to >10%) | 2 | 3 |
| Total Patients with MRD reported at End Induction | 9 | 9 |

**Additional file 1: Table S1. Breakdown of MRD data for study participants.**
