# Supplementary figures and images for: A multicenter, randomized study of decitabine as epigenetic priming with induction chemotherapy in children with AML
Source: Clin Epigenetics. 2017 Oct 5;9:108. doi: 10.1186/s13148-017-0411-x (PMC5629751; doi:10.1186/s13148-017-0411-x)

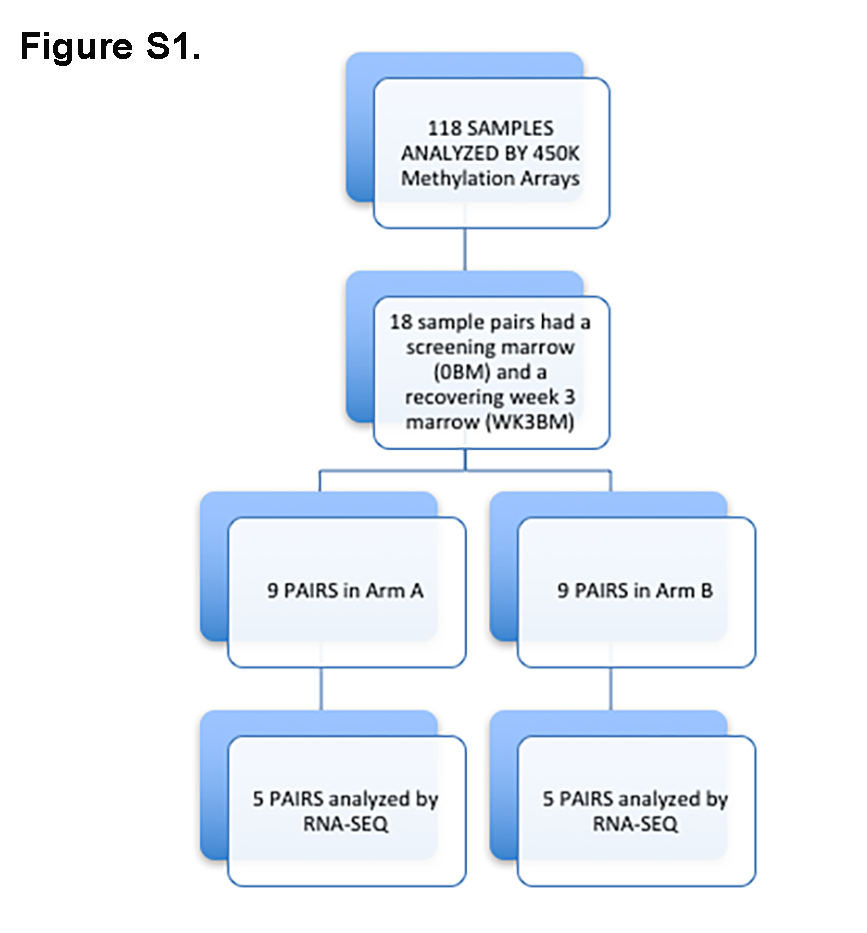

Supplement: Supplementary file 2 — Schema of sample analysis workflow. (TIFF 269 kb) [file 13148_2017_411_MOESM2_ESM.tif]

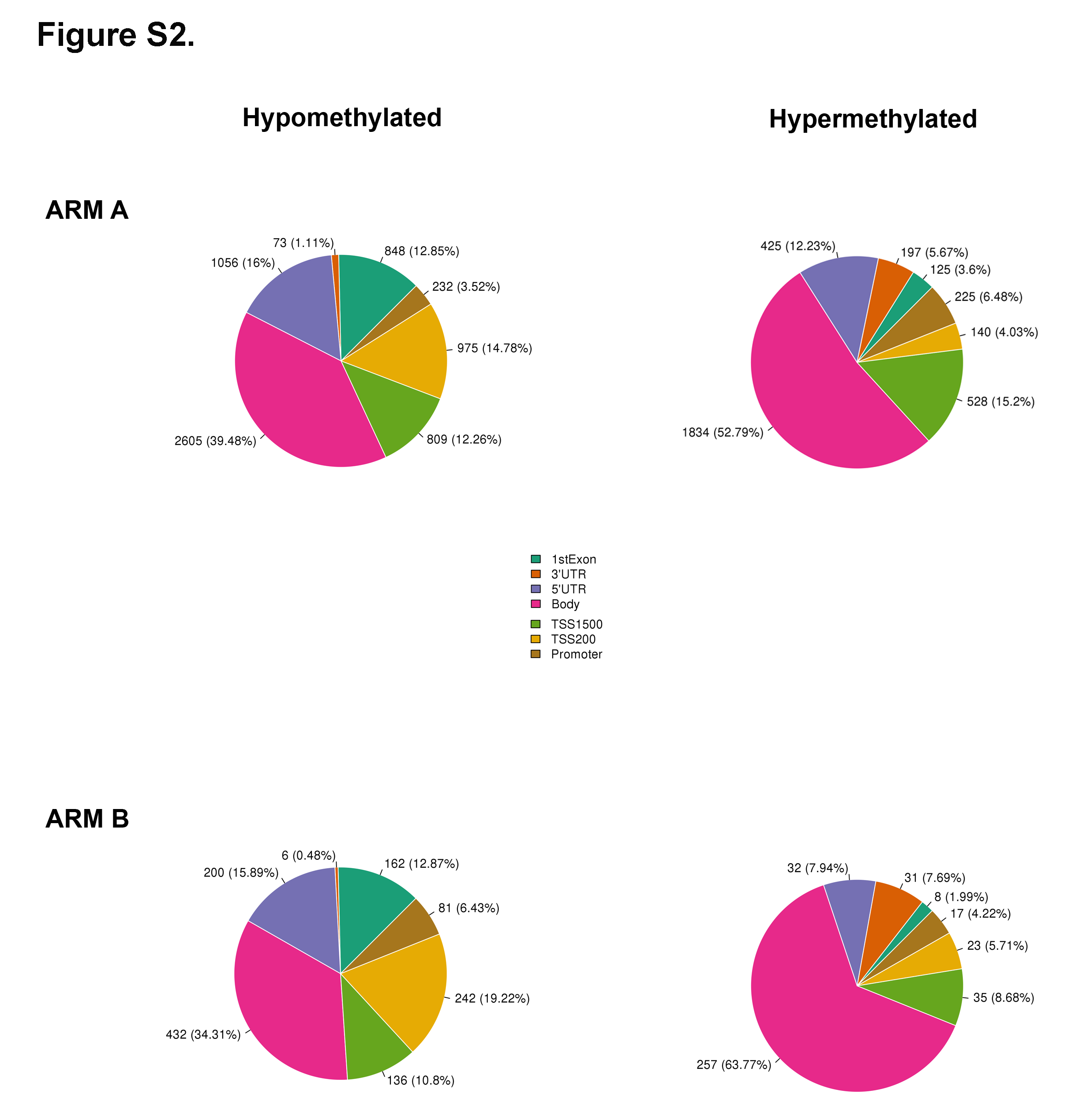

Supplement: Supplementary file 4 — Distribution of differentially methylated loci (DML) according to functional CpG contextual distribution in Arms A (decitabine + chemotherapy) and B (chemotherapy alone). Pie charts demonstrate the frequency by which hyper or hypomethylated loci are distributed according to their functional position. (TIFF 443 kb) [file 13148_2017_411_MOESM4_ESM.tif]

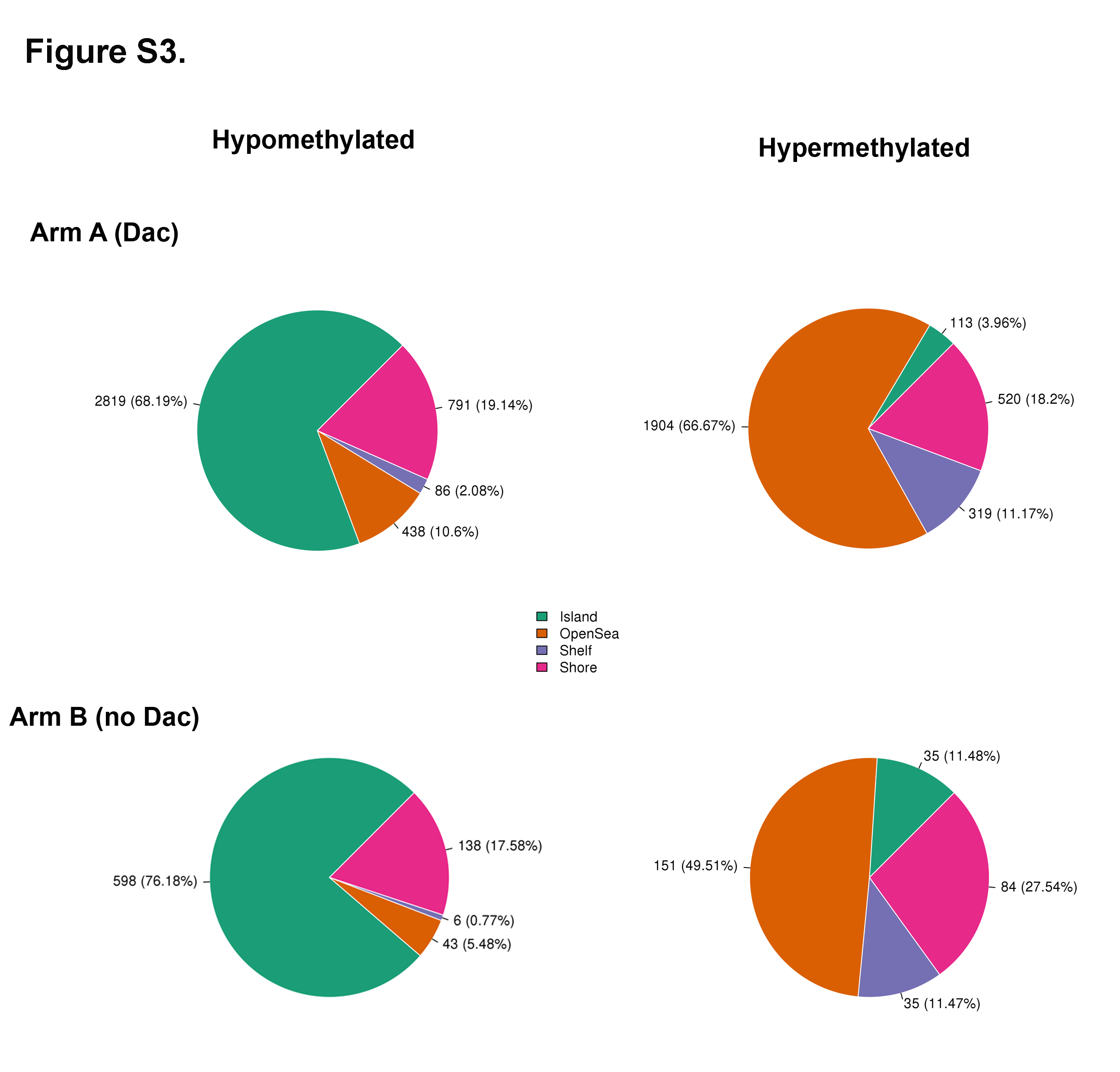

Supplement: Supplementary file 5 — Distribution of differentially methylated loci (DML) according to CpG Island contextual distribution in Arms A (decitabine + chemotherapy) and B (chemotherapy alone). Pie charts demonstrate the frequency by which hyper or hypomethylated loci are distributed according to their proximity of CpG islands. (TIFF 365 kb) [file 13148_2017_411_MOESM5_ESM.tif]

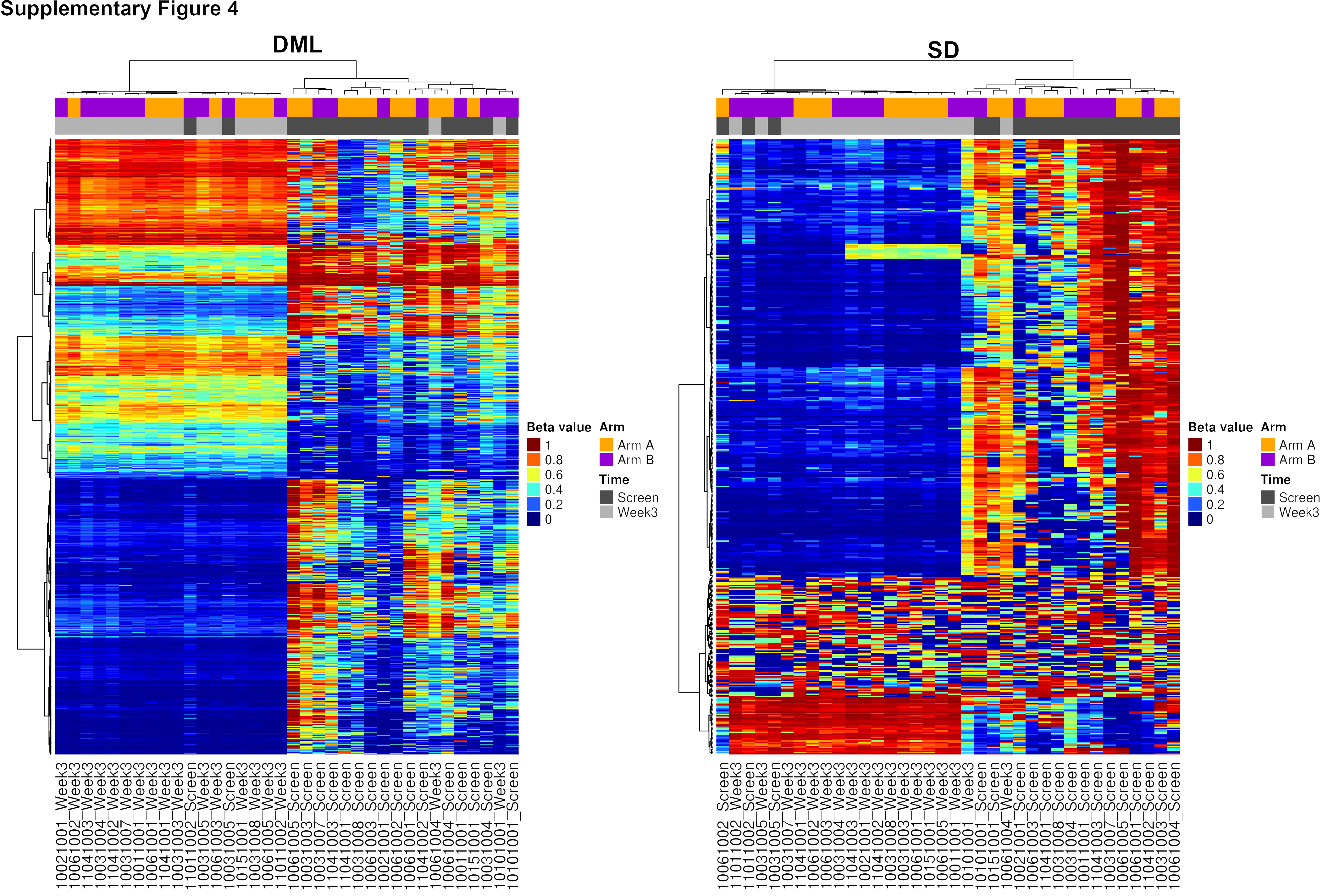

Supplement: Supplementary file 6 — Left Panel: Unsupervised hierarchical clustering of the union of differentially methylated loci (DML) for all samples in Arm A (decitabine + chemotherapy) and Arm B (chemotherapy alone). Right Panel: Unsupervised hierarchical clustering of the top 0.1% most variable loci by standard deviation for all samples in both arms. (PNG 619 kb) [file 13148_2017_411_MOESM6_ESM.png]
